# Outpatient parenteral antimicrobial therapy (OPAT) in a safety-net hospital: Opportunities for improvement

**DOI:** 10.1017/ash.2023.364

**Published:** 2023-09-29

**Authors:** Rory Bouzigard, Mark Arnold, Jacob Player, Norman Mang, Michael Lane, Trish Perl, Laila Castellino

## Abstract

**Background:** Parkland Health is a 900-bed safety-net hospital that serves Dallas County, Texas. It has an OPAT program in which patients are managed via self-administration (S-OPAT), home-health/hemodialysis (H-OPAT), and skilled nursing facilities (SNF-OPAT). We evaluated the reasons for unscheduled emergency department (ED) visits by patients in these groups to identify strategies to decrease unexpected healthcare utilization and to improve safety. **Methods:** We performed a retrospective chart review of all adult patients discharged from Parkland Health on OPAT between April and June 2021. Demographic, medical, and healthcare utilization information, including the date and reason of first unscheduled ED visit after discharge, was collected utilizing a standardized instrument. The institutional review board approved this study. **Results:** In total, 184 patients were discharged with OPAT. Among them, 32% were female and 55% identified as Hispanic; 41% were non-English speakers, and 45% were treated for a musculoskeletal infection. Among all OPAT models of care, 43.4% were S-OPAT patients, 31.5% were H-OPAT patients, and 25% were SNF-OPAT patients (Table 1). The groups differed, and fewer African Americans received H-OPAT. Also, 45% were being treated for musculoskeletal infections and were more likely to be discharged with H- or SNF-OPAT. In addition, 41% were being treated for endovascular infections and 21.7% were being treated for genitourinary infections. The total length of stay in the hospital was longer for SNF-OPAT patients and shorter for S-OPAT patients (Table 2). Among 184 OPAT patients, 41 patients (22.2%) had an ED visit: 17.3% SNF-OPAT patients, 27.6% H-OPAT patients, and 21.3% S-OPAT patients (Table 2). ED visits were attributed to intravenous (IV) access–related problems (12 of 41, 29.0%), worsening of known infection (3 of 41, 7.3%), and abnormal blood test results (2 of 41, 4.9%). Also, 24 ED visits (58%) were not related to underlying infection or OPAT. However, when examined by the OPAT care model, 41% of ED visits among S-OPAT patients, 20% among H-OPAT visits, and 25% among SNF-OPAT visits were related to IV access issues. Among S-OPAT ED visits pertaining to IV access, 71% were for minor issues such as dressing changes or line occlusion or malfunction. **Conclusions:** One-fifth of OPAT patients had an unscheduled ED visit, of whom 20%–41% had issues with IV access. Many of these visits could be avoided with enhanced outreach to patients discharged with OPAT and improved ambulatory capabilities to provide standard services related to maintenance of IV access.

**Figure:**
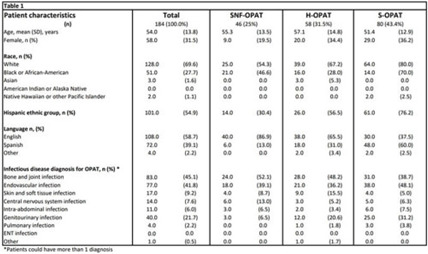

**Figure:**
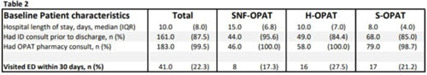

**Disclosures:** None

